# Aftershocks Associated With Impaired Health Caused by the Great East Japan Disaster Among Youth Across Japan: A National Cross-Sectional Survey

**DOI:** 10.2196/ijmr.2585

**Published:** 2013-12-20

**Authors:** Takashi Sugimoto, Tomohiro Shinozaki, Yuki Miyamoto

**Affiliations:** ^1^Graduate School of MedicineDepartment of Psychiatric NursingThe University of TokyoTokyoJapan; ^2^Graduate School of Public PolicyDepartment of Health Technology Assessment and Public PolicyThe University of TokyoTokyoJapan; ^3^Graduate School of MedicineDepartment of BiostatisticsThe University of TokyoTokyoJapan

**Keywords:** earthquakes, health communication, Internet, nuclear power plants, risk management

## Abstract

**Background:**

The Great East Japan earthquake, subsequent tsunamis and the Fukushima nuclear incident had a tremendous impact on Japanese society. Although small-scale surveys have been conducted in highly affected areas, few have elucidated the disaster’s effect on health from national perspective, which is necessary to prepare national policy and response.

**Objective:**

The aim of the present study was to describe prefecture-level health status and investigate associations with number of aftershocks, seismic intensity, a closer geographical location to the Fukushima Nuclear Power Plant, or higher reported radiation dose in each prefecture even after adjusting for individual socioeconomic factors, by utilizing individual-level data acquired from a national cross-sectional Internet survey as well as officially reported prefecture-level data.

**Methods:**

A Japanese government research institute obtained 12,000 participants by quota sampling and 7335 participants were eligible for the analysis in an age range between 17 and 27 years old. We calculated the percentage of people with decreased subjective health in each prefecture after the earthquake. Variability introduced by a small sample size for some prefectures was smoothed using empirical Bayes estimation with a random-intercept logistic model, with and without demographic factors. Multilevel logistic regression was used to calculate adjusted odds ratios (ORs) for change of subjective health associated with prefecture-level and individual-level factors.

**Results:**

Adjusted empirical Bayes estimates were higher for respondents commuting in the northeast region (Iwate 14%, Miyagi 19%, and Fukushima 28%), which faces the Pacific Ocean, while the values for Akita (10%) and Yamagata (8%) prefectures, which do not face the Pacific Ocean, were lower than those of Tokyo (12%). The values from the central to the western region were clearly lower. The number of aftershocks was coherently associated with decreased health (OR 1.05 per 100 times, 95% CI 1.04-1.06; *P*<.001) even after adjusting for covariates (OR 1.02 per 100 times, 95% CI 1.00-1.05; 1.32 per 1000 times, 95% CI 1.03-1.71; *P*=.049). In contrast, seismic intensity of the initial earthquake (OR 0.87, 95% CI 0.65-1.17; *P*=.36), radiation dose (OR 1.16, 95% CI 0.82-1.64; *P*=.41), and distance from the Fukushima Nuclear Power Plant (OR 1.00, 95% CI 0.99-1.00; *P*=.66) were not. Change in job condition (OR 2.05, 95% CI 1.72-2.45; *P*<.001), female (OR 1.43, 95% CI 1.19-1.69; *P*<.001), higher age (OR 1.06 per year, 95% CI 1.02-1.11; *P*=.005), and duration of evacuation longer than 4 weeks (OR 1.44, 95% CI 1.06-1.97; *P*=.02) seemed to decrease perceived health status.

**Conclusions:**

We found nationwide differences that show decreased health status because of the Great East Japan disaster according to prefecture. The number of aftershocks, change in work conditions, being female, a higher age, and duration of the evacuation were risk factors for the population after the major earthquake, tsunamis, and nuclear incident.

## Introduction

The Great East Japan earthquake (Tohoku earthquake) on March 11, 2011 and the subsequent tsunami had a tremendous impact on Japanese society [1]. Furthermore, the Fukushima nuclear accident, ranked 7, which is the most severe on the International Nuclear Event Scale, forced large numbers of people and industries to evacuate the area [2,3]. The explosion at the Fukushima Nuclear Power Plant (NPP) released substantial amounts of radioactive materials into the sea [4] and into the atmosphere, resulting in higher levels of radiation reaching as far as the Tokyo metropolitan area, which is 200-300 km away from the Fukushima NPP [5]. A record number of 3945 aftershocks during March after the initial earthquake and nearly 10,000 aftershocks over the following 2 years have been a stark feature of the disaster [6,7]. The persistent aftershocks and lack of credible information provided by authorities of the national government caused distress among people across Japan [8-10]. Despite such concern about health nationwide, there were no rapid assessments of nationwide health status and implementation of strategic announcements, partly because of the logistical challenges involved, but mainly because of the absence of preparedness and coordination between the central and local governments, medical communities, nongovernmental organizations, and volunteer groups [1]. Assessing public health status promptly across a nation is of major relevance to health policy decision makers as well as researchers looking at disasters.

Unlike impromptu and unsystematic surveys on health, many younger persons tried to empower others in disaster-struck areas promptly through the use of Internet technologies. Physicians and hospital officials in affected areas reported their medical resource status utilizing email lists or social media such as Twitter, which made a definite difference in the disaster response compared with the Great Hanshin earthquake of 1995 [11]. Information on bed availability and so on was collected and became available to the public within the first 48 hours of the earthquake using Google Map technology [11]. Furthermore, use of geographical information system facilitated deployment of medical teams because it provided data on radiation risks in Fukushima promptly [12]. Many younger persons, who were in their early 40s or younger who were not health care professionals, also took the initiative to gather and diffuse relevant information, garner support, and raise money for quake relief [1,13]. Substantially, many people throughout the nation used the Internet to seek accurate information and to also access the mass media. This was in some ways similar to the public health emergency response in the United States after the 9/11 attacks [1,11-14]. The Internet has now been adopted widely enough in Japanese society, especially in the younger generation cohort, to be a valuable assessment tool in a public health emergency.

Public health assessments as well as constructive advice via the Internet have unique advantages in terms of a more rapid and broader reach [1,11,13,15,16]. Specifically, surveys via the Internet are able to reach more citizens distant from the devastated area, as well as those in highly affected areas, or those with mild health complaints who have not visited a medical facility [15,16]. Most previous studies regarding the effects of the disaster on health have actually been limited to mostly severely affected areas in northeastern Japan, except for a few studies focusing on distance from the epicenter [8,17] and the effect of aftershocks on psychological stress [8]. Most previous studies limited attention to presumed high-risk populations within a highly affected area. For example, previous studies included those with cardiovascular diseases [18,19] and diabetes mellitus [20] or other diseases [21-23], the elderly [24-26], evacuees [24-26], children [27,28], workers [29-31], nonprofessional volunteers [32], caregivers [33], and pregnant women [34] in a disaster area. The limited attention seems to have been common among disaster studies. One study of the previous Niigata-Chuetsu earthquake in 2004 showed that being female had a higher odds ratio of psychological distress [35]. Furthermore, studies showed the importance of preventing mothers from having symptoms of psychological distress caused by anxiety about the health of their children and separation from family members [36-38]. We believed that the inherent restricted nature of surveys after a disaster in Japan is attributed to the scarce knowledge of potential effects on nationwide health status after a massive disaster. This would complicate a coordinated national response to a disaster.

Although the elderly or children may be the most vulnerable, a previous study indicated disruption of work after natural disaster as being independently associated with decreases in general mental and physical health among university students [39]. Another recent report revealed a significant regional difference in the perception of risk among Japanese university students in a severely hit region, an indirectly affected Tokyo region, and mostly unaffected western regions [40]. Students in the Tokyo region were anticipated to be at increased risk of a future earthquake compared with those in a victimized region as well as western Japan, although the perceived risk of further nuclear risks was approximately the same for these regions [40]. In the case of the Chernobyl disaster, people living relatively far from the disaster site tended to be more concerned about the political and economic situation [41]. To our knowledge, there are few studies on health nationwide that examine geographical factors as well as social factors after large-scale disasters such as that in Japan. Because the Tohoku disaster may have affected nationwide health in every age range due to its nuclear radiation release, persistent aftershocks, widespread concern, and expected differences in these factors among regions [1-10,17,40,41], we wanted to evaluate its impact nationwide, especially among those in their late teens or 20s who typically are seen as less vulnerable. These data are crucial for national planning, programs and recovery.

We hypothesized that there are positive associations between the decreased subjective health of the young population nationwide and a larger number of aftershocks, a closer geographical location to the Fukushima NPP, or a higher reported radiation dose in the atmosphere in each region even after adjusting for individual socioeconomic factors. Therefore, the aim of the present study is to describe prefecture-level health status and investigate the associations mentioned above after adjusting for individual socioeconomic factors, utilizing individual-level data acquired from a national cross-sectional survey as well as officially reported prefecture-level data.

## Methods

### Data Acquisition

The data for this secondary analysis, an Internet survey on the effects of the Great East Japan Disaster on career and wage among a young generation (2012), were provided by the Social Science Japan Data Archive, Centre for Social Research and Data Archives, Institute of Social Science, The University of Tokyo. The Internet survey was conducted in January 2012 to investigate the short-term effect of the Great East Japan earthquake on the wages of college or high-school graduates focusing on the role of the quality of education, by the Economic and Social Research Institute, Cabinet Office, Government of Japan [42]. The survey was conducted according to ethical guidelines for social science research and study participation was voluntary. We did not apply for a research approval from an ethics committee because this secondary analysis used data edited by the Social Science Japan Data Archive that offers archived data to academic researchers and students without any ethical or financial requests. The data had been carefully edited so it was impossible to identify individuals from any analyses.

### Participants

The survey recruited 12,000 young voluntary participants of a major Internet service in Japan based on quota sampling method [42]. The Economic and Social Research Institute reported that they followed this method rather than random sampling methods due to advantages in rapid assessment, and because random sampling does not always secure valid study participants [42]. The survey subjects graduated from college or high school between March 2009 and March 2011 across Japan, with a resultant age range of 17-27 years old. In this age range, there were 1.2 to 1.5 million peers in each year and 5% more females than males [43]. Consequently, we obtained 7335 participants after we removed participants with data missing for the prefectures in which they commuted. The graduation rates were 23.0% in 2009, 22.1% in 2010, 18.8% in 2011, and 19.1% in 2012. The remaining 17.1% of respondents had graduated from high school and would graduate from college or other schools in 2013 or later. The duration of employment after graduation varied from 0 (25%) to more than 51 months (2.6%), with a triple peak at 10 (10.2%), 22 (7.2%), and 34 months (5.5%) because the survey was conducted in January, taking into account the typical start of the fiscal year for businesses in April.

Approximately 93% of respondents did not report clearly defined adverse effects caused by the disaster. A small percentage of these participants experienced the loss of second-degree relatives (0.3%) and 0.3% experienced injuries caused by the disaster. Other adverse events included collapse of their house or official evacuation because of the crisis at the Fukushima NPP.

### Perceived Health Status

The survey asked questions directly related to the disaster. The data included a change of self-perceived health status after the disaster. The question asked was, “Did your health status change because of the Great East Japan Disaster?” There were seven categories for answers: highly improved, improved, relatively improved, unchanged, relatively decreased, decreased, and highly decreased. We categorized these seven categories into two, not-decreased and decreased, because we intended to focus on the binary difference between health and poor health. Furthermore, few respondents answered “highly improved” and “improved,” so the two categories seemed to provide a valid comparison. This binary health status change was set as the outcome variable.

### Prefecture-Level Predictors for Health Status

We categorized geographic location based on prefectures where respondents commuted according to the values assigned by the Japan Meteorological Agency Seismic Intensity scale (JMA-SI), 0-7, and the radiation dose published by the Ministry of Education, Culture, Sports, Science and Technology (MEXT) of Japan [6,44]. When levels of JMA-SI were recorded in a prefecture, we assigned the largest value as the indicator. The value of the radiation dose was considered a continuous variable, which was determined on March 20, 2011 when it was first made available by MEXT and on January 1, 2012 when the survey was conducted [44]. We calculated the distance in a straight line between each capital city of a prefecture and the Fukushima NPP and included the number of aftershocks from March 11, 2011 to January 31, 2012 recorded in the JMA database [7].

### Six Area Indicators in Japan

**Figure 1:**
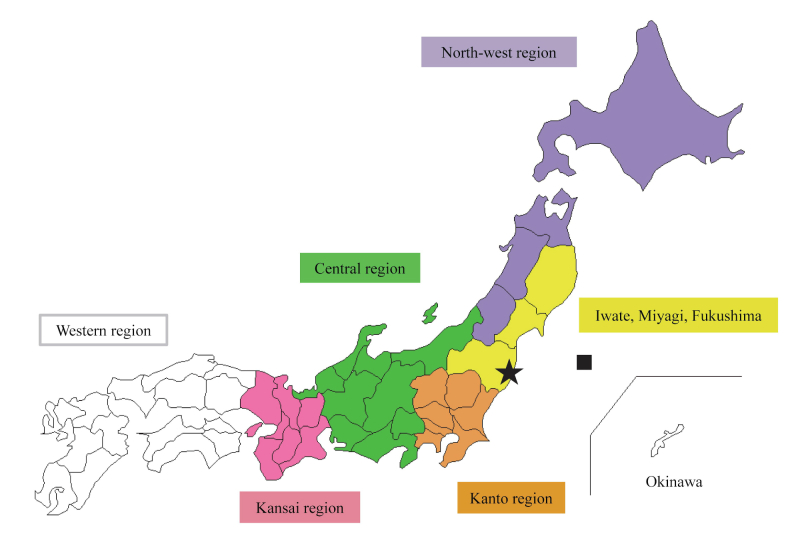
Map of Japan divided into six area indicators. The black square indicates the epicentre of the earthquake. The black star indicates the location of the Fukushima NPP.

We introduced six area indicators: Northwest region; Iwate, Miyagi, and Fukushima; Kanto region; Central region; Kansai region; and Western region as shown in Figure 1. The Northwest region was an indirectly affected region because it was within the Tohoku area; however, it did not suffer at all from the tsunami and was relatively distant from the Fukushima NPP. Iwate, Miyagi, and Fukushima were directly affected regions because these three areas were most affected by the tsunami, the Fukushima incident, and persistent aftershocks. The Kanto region was an indirectly affected region because it did not bear much of the impact from the tsunami, but suffered from persistent aftershocks as well as anticipation about radiation. The Central and Kansai regions were indirectly affected regions that are relatively distant from Fukushima, but nearer compared to western Japan. The Western region was set as the control group because they were distant from Fukushima and had fewer aftershocks.

### Individual-Level Demographic Factors

Marital status was based on current status, so that widows or divorcées were classified as not married. These participants were categorized in the following manner: never married, 94.1%; married, 5.8%; widows, 0.1%; and divorced, 0.2%. Respondents were asked to answer “No” to the questions about change in employment if the corresponding conditions were caused by intentional career changes or other personal reasons. If a person had lived apart from other family members before the disaster, respondents were asked to answer “No” to questions about separation from their families. A change of working conditions included a reduction of sales (5.1%), reduction and upgrade of graduate recruitment (2.9%), merger and acquisition (0.8%), attrition (1.8%), damage of plants or capital (7.2%), temporary suspension of business (8.1%), and reduction of compensation (2.1%). Participants answered only when the condition was true. Individual economic status was assessed according to income. A difference in income between in 2010 and after the disaster in 2011 was categorized as “minus 2 levels” or “plus 1 level,” where a unit of “level” represented approximately one million yen. In contrast, an expected decrease of income in the 2012 fiscal year was answered yes or no.

### Statistical Analysis

We calculated the percentage of people with decreased self-perceived health status in each prefecture. Because sampling error from the Internet survey seemed to render the percentages highly variable due to the small sample size in individual prefectures, we smoothed the actual percentages with empirical Bayes predictions from the multilevel (mixed effects) logistic regression by introducing random intercepts for 47 prefectures and an overall constant (the mean across all clusters/population mean) [45]. The empirical Bayes predictions can effectively “shrink” the actual percentages to a population mean, according to each prefecture’s size [46]. To adjust for differences in individual demographic factors between prefectures, we also fitted the model with gender, age, education, marital and employment status, changed job condition, income, death of family member[s], being a parent, family separation, and evacuation. Consequently, the prediction for each prefecture is presented by a regression standardization method [47]. In theory, regression standardization estimates the expectation of an outcome had all individuals been assigned a variable of interest at the specified level (an index level). Because the variable of interest in the analysis was a prefecture membership (incorporated as random intercepts) and index levels were the 47 indicators of prefectures in Japan, we presented the adjusted empirical Bayes predictions for 47 prefectures as the sum of the random intercepts and the mean of 7335 predictions from fixed parts of the model (ie, individual demographic factors) averaged over the whole population, followed by expit transformation: exp(*η*+ *γ*_*i*_)/[1 + exp(*η*+ *γ*_*i*_)] for mean linear predictior *η* of fixed effects and a random effect *γ*_*i*_ in prefecture *i* (*i*=1,…, 47). Because expit of mean of individual predictiors differs from mean of expit of individual predictiors, our adjusted empirical Bayes predictions approximated the standardized percentages in first order.

To identify a regional association between the change of self-perceived health status and environmental factors (prefecture-level variables), the odds ratios (ORs) and their 95% CIs were calculated from fixed-effects logistic models. Predictor variables included (a) radiation dose, distance from the Fukushima NPP, JMA-SI, and number of aftershocks from March 11, 2011 to January 31, 2011 with or without (b) the area indicators in Japan. We calculated crude and adjusted ORs from univariable and from multivariable-adjusted logistic models, respectively. Model 1 included prefecture-level variables (a), Model 2 included the area indicator (b), and Model 3 simultaneously introduced all prefecture-level variables (a) and (b). We fitted different models for sensitivity analysis rather than for model building with a rejection of unnecessary covariates, although Akaike's Information Criterion (AIC, which measures prediction error by estimating the mean of Kullback-Leibler divergence over asymptotic sampling distributions) was presented not so as to search for an accurate prediction, but just as a reference for readers.

To simultaneously estimate the association of prefecture-level variables and demographic variables (individual-level variables), including loss of family, changes in work conditions, individual economic status, experience of evacuation and separation from family, to self-perceived health status, we fitted multilevel logistic regression models that included random-intercepts for 47 prefectures and the above prefecture- and individual-level variables. The sensitivity of individual-level effects to adjustment for prefecture-level variables was analysed by fitting different models conducted in the same manner as above: Model 1 included individual-level variables only; Model 2 adjusted Model 1 with the prefecture-level variables (a); and Model 3 simultaneously adjusted for prefecture-level variables (a) and area indicators (b).

The variables included in the models were selected from a questionnaire regarding the existing literature, which investigated the effects of the disaster on subsequent distress. Because of unclear previous knowledge on interactions, we did not conduct stratified analyses and did not include interaction terms in the multivariable models. Conformity with a linear gradient in the model was checked graphically before fitting regression models. All statistical and graphical analyses were conducted using R version 3.0.1 for Windows. The lme4 and glmmML packages were primarily used.

## Results

### Overview

Respondents’ demographic characteristics are shown in Table 1. Those with decreased perceived health status tended to include more females, slightly more often changed their job condition, left their family member(s) for a longer duration, and had evacuated longer than those without decreased perceived health status.

### Prefecture-Level Changes in Health Status

The decrease in self-perceived health status differed significantly among respondents in the prefectures as shown in Table 2.

Compared with Tokyo, respondents commuting in Miyagi and Fukushima prefectures, which are located adjacent to Fukushima, showed a statistically significant decrease in health status. Respondents commuting in Iwate and Tochigi prefectures also reported an increased reduction in health status, although the differences were not statistically significant. The values were higher for respondents commuting in the Tohoku area (Iwate, Miyagi, and Fukushima), which faces the Pacific Ocean, while the values for Akita and Yamagata prefectures, which do not face the Pacific Ocean, were lower than those of Tokyo. In contrast, there were many prefectures where respondents’ health status was less likely to be reduced, particularly in Hokkaido and the central and western regions of Japan. In addition to those in Tokyo, those commuting in Okinawa, Kochi, and Toyama prefectures also reported a high frequency of decreased health status. In general, young people commuting in Tokyo reported a relatively higher reduction of their perceived health status compared with those commuting in many western regions of Japan.

**Table 1:** Demographic characteristics by the change of subjective health.

		Perceived health status	
Variables		Decreased (n=649)	Not decreased (n=6686)
		n (%) or mean (SD)	n (%) or mean (SD)
**Gender, n (%)**			
	Male	262 (40.37)	3097 (46.32)
	Female	387 (59.63)	3589 (53.68)
**Age, n (%)**			
	17 years old	4 (0.62)	49 (0.73)
	18 years old	5 (0.77)	107 (1.60)
	19 years old	14 (2.16)	196 (2.93)
	20 years old	24 (3.70)	372 (5.56)
	21 years old	38 (5.86)	459 (6.87)
	22 years old	77 (11.86)	794 (11.88)
	23 years old	107 (16.49)	984 (14.72)
	24 years old	127 (19.57)	1363 (20.39)
	25 years old	132 (20.34)	1269 (18.97)
	26 years old	67 (10.32)	623 (9.32)
	27 years old	54 (8.32)	470 (7.03)
	Mean age (SD)	23.74 (2.04)	23.48 (2.17)
**Education, n (%)**			
	College students	462 (71.19)	4701 (70.31)
	Not college students	187 (28.81)	1985 (29.69)
**Marital status, n (%)**			
	Married	42 (6.47)	390 (5.83)
	Not married	607 (93.53)	6296 (94.17)
**Employment status, n (%)**			
	Regular employee	248 (38.21)	2375 (35.52)
	Not regular employee	401 (61.87)	4311 (64.48)
**Changed job condition, n (%)**			
	Yes	245 (37.50)	1371 (20.51)
	No	404 (62.25)	5315 (79.49)
Difference of income 2011–2010^a^, mean (SD)		0.27 (1.50)	0.20 (1.38)
**Expected income in 2012, n (%)**			
	Will be decreased	45 (6.93)	361 (5.40)
	Will be increased/stable	604 (93.07)	6325 (94.60)
**Number of deaths of family members, n (%)**			
	> 1	3 (0.46)	16 (0.24)
	0	646 (99.54)	6670 (99.76)
**Having a child/children, n (%)**			
	Yes	24 (3.70)	263 (3.93)
	No	625 (96.30)	6423 (96.07)
**Duration left for the family member(s), n (%)**			
	> 4 weeks	66 (10.17)	396 (5.92)
	≤ 4 weeks	583 (89.83)	6290 (94.08)
**Duration of evacuation, n (%)**			
	> 4 weeks	91 (14.02)	528 (7.90)
	≤ 4 weeks	558 (85.98)	6158 (92.10)

^a^Change of categorical level, where a unit of “level” represented approximately one million yen. Example: difference is −2 when the level was 5 in 2010 and 3 in 2011.

**Table 2:** Commuting location and the decreased self-perceived health.

	JMA-SI (level) ^a^	Radi (µSv/h)	Quake (times)	Decreased (n)	Not decreased (n)	Decreased (%)	*P* value
Miyagi	7	0.111^b^	2841	28	93	23.1	.002^d^
Fukushima	6	2.5^c^	4211	26	44	37	<.001^e^
Tochigi	6	0.154	1552	11	43	20	.09
Iwate	6	0.027	2304	9	40	18	.19
Ibaraki	6	0.176	3422	16	78	17	.20
Aomori	5	0.02	818	8	36	18	.25
Tokyo	5	0.048	691	232	1643	12.37	Reference
Chiba	5	0.033	1526	22	176	10.9	.73
Akita	5	0.034	644	3	26	10	1.00
Saitama	5	0.057	946	21	187	10.1	.37
Nagano	5	0.069	868	8	73	10	.60
Kanagawa	5	0.049	435	40	387	9.4	.09
Yamagata	5	0.04	862	3	33	8	.61
Gunma	5	0.08	939	6	75	7	.22
Niigata	5	0.047	817	6	82	7	.13
Yamanashi	5	0.044	262	2	30	7	.42
Gifu	4	0.062	212	9	106	7.8	.18
Hokkaido	4	0.027	395	17	229	6.9	.01^f^
Shizuoka	4	0.037	371	11	172	6.0	.008^d^
Aichi	4	0.041	79	19	533	3.4	<.001^e^
Toyama	3	0.047	77	8	50	14	.69
Shiga	3	0.034	57	7	56	11	1.00
Nara	3	0.048	42	5	61	8	.34
Fukui	3	0.045	54	3	47	6	.27
Hyogo	3	0.037	47	18	290	5.8	<.001^e^
Kyoto	3	0.039	43	12	208	5.5	.002^d^
Mie	3	0.046	38	5	87	5	.048^f^
Osaka	3	0.043	43	31	693	4.3	<.001^e^
Ishikawa	3	0.046	88	2	52	4	.056
Shimane	2	0.036	38	3	31	9	.79
Wakayama	2	0.032	92	4	45	8	.51
Okayama	2	0.049	31	5	109	4.4	.007^d^
Totori	2	0.063	21	0	28	0	.04^f^
Tokushima	2	0.039	29	0	41	0	.007^d^
Kochi	1	0.026	33	4	26	13	.78
Kagawa	1	0.053	21	3	40	7	.36
Nagasaki	1	0.029	21	3	40	7	.36
Fukuoka	1	0.037	25	14	224	5.8	.002^d^
Oita	1	0.05	39	2	39	5	.22
Hiroshima	1	0.05	52	7	147	4.5	.002^d^
Kumamoto	1	0.027	75	2	49	4	.08
Kagoshima	1	0.035	130	1	41	2	.053
Ehime	1	0.047	28	1	60	2	.007^d^
Saga	1	0.04	12	0	19	0	.16
Okinawa	0	0.021	56	5	29	15	.60
Miyazaki	0	0.027	44	3	24	11	1.00
Yamaguchi	0	0.094	21	4	61	6	.17

^a^JMA-SI, Japan Meteorological Agency seismic intensity; Radi, radiation dose on March 20, 2011 (µSv/h); Quakes, total number of aftershocks from March 11, 2011 to January 31, 2012.

^b^Obtained initially at 19:00 on March 29, 2011.

^c^Obtained initially at 13:00 on April 6, 2011.

^d^Mean < .01.

^e^Mean <.001.

^f^Mean < .05.

### Empirical Bayes Predictions of the Percentages of Decreased Health Status

**Figure 2:**
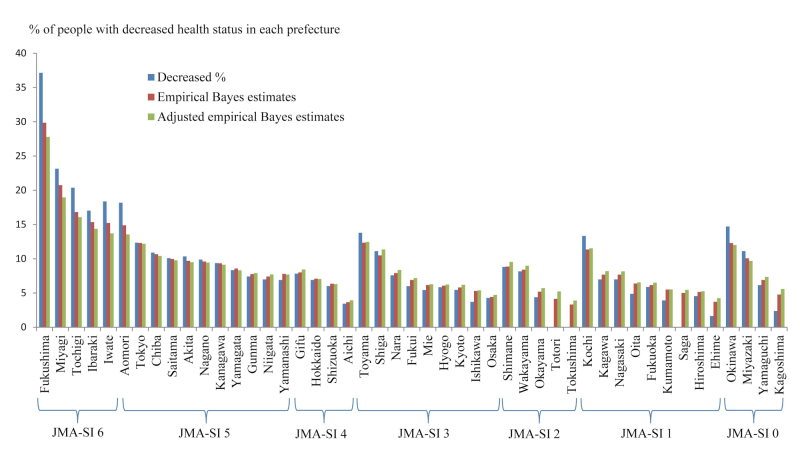
Percentage of respondents reporting decreased self-perceived health status and empirical Bayes estimates in each prefecture. JMA-SI, Japan Meteorological Agency Seismic Intensity. For the same JMA-SI levels, we determined the rank order of prefectures based on the values of adjusted empirical Bayes estimates. In adjusted empirical Bayes estimates, percentages were also adjusted according to demographic factors (gender, age, education, marital and employment status, changed job condition, income, death of family member[s], being a parent, family separation, and evacuation).

Empirical Bayes estimates from random-effects logistic models of each prefecture’s proportion of respondents with decreased self-perceived health status, as well as the actual percentages from Table 2 are plotted in Figure 2 according to JMA-SI level.

**Figure 3:**
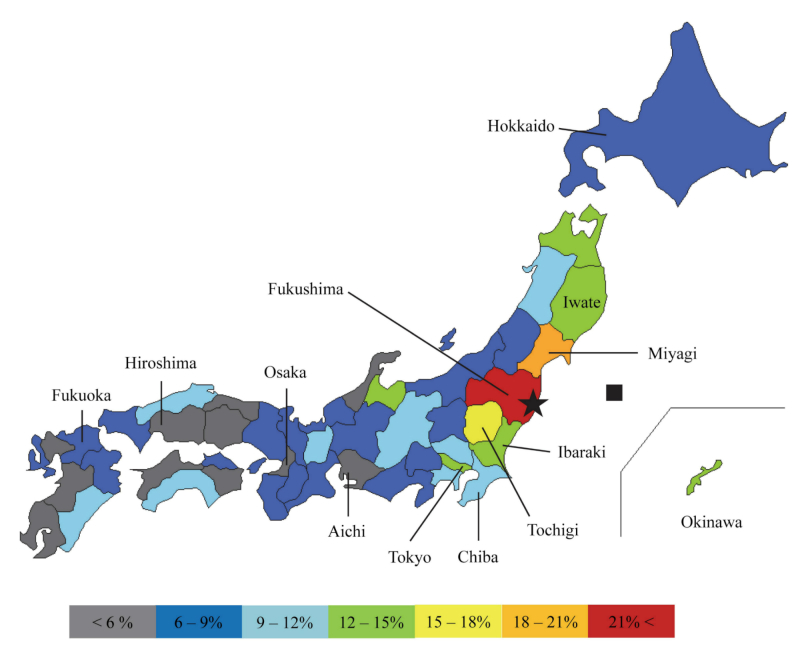
Map of Japan depicting adjusted empirical Bayes estimates for percentage of people with decreased self-perceived health status. Red (>21%), orange (18%–21%), yellow (15%–18%), chartreuse green (12%–15%), aquamarine (9%–12%), blue (6%–9%), and gray (<6%). The black square indicates the epicenter of the earthquake. The black star indicates the location of the Fukushima NPP.

The percentages of respondents commuting in Tochigi and Ibaraki prefectures as well as Iwate, Miyagi, and Fukushima are the highest. Surprisingly, the percentages for respondents commuting in Tokyo and Chiba are higher compared with the rest of Japan. In contrast, the percentage of reports of decreased health status from central to the western region (Aichi, Osaka, and prefectures located at more western than them) is clearly lower (Figure 3).

### Prefecture-Level Factors Associated With Decline in Health Status

The regional association between the change of self-perceived health status and prefecture-level variables estimated from fixed-effects logistic models are depicted in Table 3. From Model 1, the total number of quakes (OR 1.03, 95% CI 1.01-1.05; *P*<.001) and the seismic intensity of the primary quake (OR 1.18, 95% CI 1.08-1.29; *P*<.001) were associated with the prevalence of the decreased self-perceived health, but after adjustment for regional areas (Model 3), the association of seismic intensity vanished (OR 0.90, 95% CI 0.68-1.20; *P*=.48). On the other hand, the area of Iwate, Miyagi and Fukushima (OR 6.29, 95% CI 4.25-9.32; *P*<.001 in Model 2) was strongly associated with decreased self-perceived health even after adjustment for the prefecture-level factors in Model 3 (OR 4.63, 95% CI 1.10-19.57; *P*=.04).

The results from random-intercept multilevel logistic models for the probability of decreased self-perceived health status including individual-level demographic variables and/or prefecture-level variables are presented in Table 4. Each model provided essentially the same results. Change in job condition (OR 2.05, 95% CI 1.72-2.45; *P*<.001), female (OR 1.43 [=1/0.70, inverse of OR for male 0.70, 95% CI 0.59-0.84]; *P*<.001), higher age (OR 1.06 per year, 95% CI 1.02-1.11; *P*=.005), and duration of evacuation longer than 4 weeks (OR 1.44, 95% CI 1.06-1.97; *P*=.02) seemed to decrease perceived health status in Model 3. As in Table 3, the total number of quakes (OR 1.02 per 100 times, 95% CI 1.00-1.05; *P*=.049) was strongly associated with decreased self-perceived health after adjustment for individual-level demographic factors. The OR became 1.32 (95% CI 1.03-1.71) per 1000 times. The area of Iwate, Miyagi, and Fukushima (OR 4.45, 95% CI 0.99-20.01; *P*=.052) and Kanto region (OR 2.94, 95% CI 0.94-9.18; *P*=.06) also showed a strong association. As a reviewer pointed out, although Model 3 probably suffers from the unstability of estimates due to multiple collinearity (eg, evacuated seemed obviously a function of distance to epicenter), our models provided coherent results for these variables.

The radiation levels reported relatively soon, or considerably after the Fukushima NPP crisis and the distance from the nuclear power plant are not significantly associated with decreased health status after adjusting for covariates and potential covariates. The death of family members is not significantly associated with decreased health status because of the small number of events.

**Table 3:** Prefecture-level factors associated with decreased health status: regional-level logistic regression analysis.

		Model 1		Model 2		Model 3	
Independent variables	Crude OR^a^ (95% CI)	Adjusted OR (95% CI)	*P* value	Adjusted OR (95% CI)	*P* value	Adjusted OR (95% CI)	*P* value
From Fukushima NPP^b^ (km)	0.99 (0.99-1.00)	1.00 (0.99-1.00)	.29	–	–	1.00 (1.00-1.00)	.65
Radiation^c^ (μSv/hr)	2.09 (1.72-2.55)	1.16 (0.87-1.53)	.31	–	–	1.11 (0.81-1.51)	.51
Total quakes^d^ (x10^−2^)	1.05 (1.04-1.06)	1.03 (1.01-1.05)	<.001	–	–	1.03 (1.00-1.05)	.02
JMA-SI^e^	1.38 (1.29-1.47)	1.18 (1.08-1.29)	<.001	–	–	0.90 (0.68-1.20)	.46
Northwest region	0.98 (0.68-1.44)	–	–	1.69 (1.07-2.67)	.02	2.11 (0.80-5.58)	.13
Iwate, Miyagi, and Fukushima	3.95 (2.93-5.34)	–	–	6.29 (4.25-9.32)	<.001	4.63 (1.10-19.57)	.04
Kanto region	1.82 (1.55-2.14)	–	–	2.37 (1.77-3.16)	<.001	2.93 (0.99-8.66)	.05
Central region	0.57 (0.44-0.74)	–	–	1.05 (0.73-1.52)	.77	1.34 (0.56-3.23)	.51
Kansai region	0.53 (0.41-0.67)	–	–	1.01 (0.72-1.43)	.97	1.23 (0.65-2.32)	.53
		AIC^f^ value = 4252		AIC value = 4244		AIC value = 4241	

^a^OR, odds ratio

^b^NPP, nuclear power plant

^c^Radiation, radiation dose on March 20, 2011 (μSv/h)

^d^Total quakes, total number of aftershocks from March 11, 2011 to January 31, 2012

^e^JMA-SI, Japan Meteorological Agency seismic intensity

^f^AIC, Akaike's Information Criterion

**Table 4:** Individual- and prefecture-level factors associated with decreased health status: multilevel logistic regression analysis with random-intercept for 47 prefectures.

		Model 1		Model 2		Model 3	
Independent variables	Crude OR^a^ (95% CI)	Adjusted OR (95% CI)	*P* value	Adjusted OR (95% CI)	*P* value	Adjusted OR (95% CI)	*P* value
Regular employee (ref: not)	0.89 (0.75-1.05)	0.85 (0.71-1.01)	.07	0.85 (0.71-1.01)	.06	0.85 (0.71-1.02)	.07
Change in job condition (ref: not)	2.35 (1.98-2.79)	2.12 (1.77-2.52)	<.001	2.05 (1.72-2.45)	<.001	2.05 (1.72-2.45)	<.001
Difference in income 2010–2011 (million yen incr.)	1.03 (0.98-1.09)	1.03 (0.97-1.09)	.35	1.03 (0.97-1.09)	.34	1.02 (0.97-1.09)	.35
Income will decrease (ref: not)	1.31 (0.95-1.80)	1.31 (0.94-1.83)	.11	1.31 (0.94-1.82)	.11	1.31 (0.95-1.84)	.10
Sex (male=1, female=0)	0.78 (0.67-0.92)	0.71 (0.60-0.84)	<.001	0.71 (0.60-0.84)	<.001	0.70 (0.59-0.84)	<.001
Age (year)	1.06 (1.02-1.10)	1.06 (1.02-1.11)	.006	1.06 (1.02-1.11)	.005	1.06 (1.02-1.11)	.005
Marital status (Married = 1)	1.12 (0.80-1.55)	0.94 (0.66-1.33)	.72	0.92 (0.65-1.30)	.64	0.92 (0.65-1.30)	.63
College student (ref: not)	1.04 (0.87-1.25)	1.00 (0.82-1.22)	.99	0.99 (0.82-1.22)	.996	1.00 (0.83-1.27)	.95
Family separation (> 4 weeks; ref: not)	1.80 (1.37-2.37)	1.22 (0.85-1.74)	.28	1.21 (0.85-1.74)	.28	1.21 (0.85-1.73)	.30
Evacuation (> 4 weeks; ref: not)	1.90 (1.50-2.41)	1.48 (1.08-2.03)	.01	1.45 (1.06-1.98)	.02	1.44 (1.06-1.97)	.02
Death of family members (ref: not)	1.94 (0.56-6.66)	2.33 (0.66-8.26)	.19	2.28 (0.65-8.03)	.20	2.34 (0.66-8.26)	.19
Having a child/children (ref: not)	0.94 (0.61-1.44)	0.93 (0.60-1.44)	.75	0.94 (0.61-1.45)	.77	0.93 (0.59-1.44)	.74
From Fukushima NPP^b^ (km)	0.99 (0.99-1.00)	–	–	0.99 (0.99-1.00)	.57	1.00 (0.99-1.00)	.66
Radiation^c^ (μSv/hr)	2.09 (1.72-2.55)	–	–	1.17 (0.80-1.69)	.42	1.16 (0.82-1.64)	.41
Total quakes^d^ ( x10^−2^)	1.05 (1.04-1.06)	–	–	1.04 (1.01-1.06)	.002	1.02 (1.00-1.05)	.049
JMA-SI^e^	1.38 (1.29-1.47)	–	–	1.07 (0.96-1.20)	.24	0.87 (0.65-1.17)	.36
Northwest region	0.98 (0.68-1.44)	–	–	–	–	2.21 (0.78-6.26)	.14
Iwate, Miyagi, and Fukushima	3.95 (2.93-5.34)	–	–	–	–	4.45 (0.99-20.01)	.05
Kanto region	1.82 (1.55-2.14)	–	–	–	–	2.94 (0.94-9.18)	.06
Central region	0.57 (0.44-0.74)	–	–	–	–	1.49 (0.59-3.78)	.40
Kansai region	0.53 (0.41-0.67)	–	–	–	–	1.32 (0.66-2.62)	.43
		AIC^f^: 4185		AIC: 4155		AIC: 4158	

^a^OR, odds ratio

^b^NPP, nuclear power plant

^c^Radiation, radiation dose on March 20, 2011 (μSv/h)

^d^Total quakes, total number of aftershocks from March 11, 2011 to January 31, 2012

^e^JMA-SI, Japan Meteorological Agency seismic intensity

^f^AIC, Akaike's Information Criterion

## Discussion

### Three Novel Findings

We first illustrate a prefecture-level difference that shows decreased health status because of the Great East Japan Disaster. Our study found three novel findings. First, the present study shows a coherent association between decreased health status and number of aftershocks. Second, the prefecture-level radiation dose reported after the Fukushima Nuclear Crisis and the distance from the NPP of each prefecture are not significantly associated with decreased health status after adjusting for covariates and potential covariates. Third, we showed coherently that changes in job condition, being female, higher ages in the late teens and 20s, and duration of evacuation longer than 4 weeks were associated with a nationwide decreased health status even after adjusting for regional-level and prefecture-level variables.

### Related Factors for the Three Novel Findings

People living relatively far from the disaster site tended to be more concerned about the political and economic situation [41]. The same study indicated that concerns about future health may have stimulated greater awareness of physical sensations, according to results indicating that subjective health was partially mediated by perceptions of hazard and risk [41]. Students in the Kanto region felt that they were at higher risk of a future earthquake compared with those in Western Japan [40]. Historically, the significant number of aftershocks in eastern Japan, including the Kanto region, would make subjective health decrease via a perception of hazard and risk for future earthquakes. This assumption might be valid from a nationwide perspective because radiation dose reported at the prefecture-level and at a distance from the Fukushima NPP were not significantly associated with decreased subjective health. Prefecture-level differences in subjective health could not be simply explained by radiation dose reported or distance from the Fukushima NPP. Rather, the health of youth nationwide is affected by aftershocks and sociodemographic factors.

Consistent with a previous study [39], change in work conditions after the disaster was independently associated with decreases in the subjective health of a young cohort nationwide. Changes in work conditions, whether this was derived from natural disasters, might cause depressive symptoms, a larger burden from work, or economic decline. Being female was robustly indicated as a significantly associated factor consistent with a previous study [35], although we could not determine the reason from the present data. Surprisingly, a higher age within the age range of 17-27 years showed a significant association. We believe that our common sense tends to perceive a higher age as a risk factor for many health problems, but generally we think of this for those aged over 65 or higher. This common thinking about age range, such as those “aged 65 or older” or “children” may result in ignorance on the impact of these events at various ages. Our data regarding an association of decreased subjective health with evacuation from the disaster area must be interpreted with care. To the best of our knowledge, there has been no evidence that evacuation from a disaster area, or from Kanto region to Western region, is a risk factor for those in their late teens or 20s. Previous studies indicated evacuation as a risk factor for high mortality or hospitalization among the elderly because relocation affects living and care quality [25,48], and also the loss of their social network, which results in psychological distress [49].

### Strengths of the Study

We believe that the present study has several significant strengths. First, we considered self-perceived health, which could include both physical and psychological aspects of health. This allowed us to evaluate general health as a whole rather than focusing on specific diseases. For instance, we could include relatively moderate illnesses that would escape inclusion in studies based on hospital records. A previous study reported that people who were professionally exposed to a disaster reported more physical and mental health complaints even in the absence of abnormal clinical laboratory values [50]. Therefore, it is definitely of value to include subjective health rather than to focus only on objective clinical diagnosis even while assuming that the percentages of self-reported illness are higher than those of clinically verified illnesses as indicated by a previous study [51]. Second, we included all prefectures in Japan rather than focusing only on the devastated area. This study thus illuminates otherwise likely-to-be-missed effects on the health of a population across the nation.

### Limitations

Our study has several limitations. First, respondents may not represent the entire population of Japanese of the same age range because the data were not collected randomly. Therefore, the data may be biased toward participants possessing higher Internet literacy or health status and who are more likely to answer the questionnaire voluntarily. Despite the relatively limited target population analyzed here, we are confident that the results can be generalized to this entire age group across Japan, because of the common ability to access the Internet among those in this age range. Second, our data was derived from a cross-sectional survey, which does not allow determination of the direction of the relationships between demographic variables and self-perceived health status. Third, there is no information on specific reasons why participants answered that they were experiencing diminished health. However, the questionnaire was sufficiently well-controlled because all questions always included the criterion, “because of the disaster,” and not just “after the disaster.” Therefore, we could assume that we minimized the probability that the reported decrease in subjective health was derived from other causes. Last, one question asked study participants, “In what prefecture do you commute?” Therefore, we could not predetermine the location of residence. Because some employees or students might commute between prefectures, the distribution shown in the map of Japan may change if we specified the location of residence. Despite these limitations, the data presented here on post-earthquake subjective health status of this age group across the nation are worth reporting.

### Implications for Future Disaster Preparedness and Health Policy

Future major earthquakes may affect health among the broader population, including youth, across the nation via persistent aftershocks and other socioeconomic disruption. Assessing public health status promptly across a nation is of major relevance to health policy decision makers as well as researchers looking at disasters. Assessments via the Internet may be a better measure in public health emergencies and subsequent phases compared to traditional paper-and-pencil-based surveys, especially for subgroups accustomed to Web technologies [52]. Differences in response characteristics and optimizing designs of Web surveys have been examined in some countries [15,16,52,53]. The effects of long-term low-dose radiation exposure on health and useful measurement technologies will be further elucidated in the future [54]. Traditional surveys may suffer from recall bias and low response rates, and impede arriving at reliable results. Rapid and cost-efficient assembly of health information requires the utilization of e-health technologies as well as epidemiological insights to provide better information to all decision makers. Researchers should be cautious in continuing with unconscious old-fashioned strategies for health assessments after a disaster and should better utilize epidemiology and information technologies to further knowledge in this field.

### Conclusions

We first investigated the extent to which subjective health of participants in each prefecture across Japan decreased as a result of the Great East Japan Disaster. We found that the number of aftershocks was coherently associated with decreased subjective health. In contrast, radiation dose and distance from the Fukushima NPP were not associated. A Web-based survey can provide valuable information on public health issues after a disaster, especially if information technologies are developed that integrate with epidemiology research.

## Abbreviations

AIC: Akaike's Information Criterion
JMA-SI: Japan Meteorological Agency seismic intensity
MEXT: Ministry of Education, Culture, Sports, Science and Technology
NPP: Nuclear Power Plant
OR: odds ratio
